# Auditory illusory models as proxies to investigate bottom-up and top-down neural networks of phantom perception

**DOI:** 10.1162/imag_a_00574

**Published:** 2025-05-09

**Authors:** Feifan Chen, Anusha Yasoda-Mohan, Colum Ó. Sé, Sven Vanneste

**Affiliations:** Lab for Clinical and Integrative Neuroscience, Trinity College Institute for Neuroscience, School of Psychology, Trinity College Dublin, Dublin, Ireland; Global Brain Health Institute, Trinity College Dublin, Dublin, Ireland; Brain Research Centre for Advanced, International, Innovative and Interdisciplinary Neuromodulation, Ghent, Belgium

**Keywords:** auditory illusions, tinnitus, phantom perception, theta oscillation, connectivity

## Abstract

Auditory phantom perception, exemplified by tinnitus, is characterized by a perceptual experience without external stimuli. This study utilized two auditory illusions, the Zwicker Tone (ZT) and Conditioned Hallucinations (CH), as proxies to investigate the neural correlates of bottom-up and top-down mechanisms underlying phantom auditory perception. Using a within-subject design, ZT, driven by temporary sensory deficits, and CH, influenced by multisensory expectations, were examined in a sample of healthy participants. Electrophysiological measures revealed distinct time–frequency patterns, with increased theta activity in central regions during ZT perception but decreased parietal theta power during CH perception. Key regions in the ZT network, including the medial prefrontal cortex, lateral orbitofrontal cortex, and ventral posterior cingulate cortex, suggested the involvement of the default mode network and predictive processing in compensating for sensory deficits. In contrast, CH perception implicated the parahippocampus, entorhinal cortex, and inferior temporal gyrus in modulating multisensory associations and cognitive expectations. Taken together, this study revealed the neural mechanism of two auditory illusions, which enhances understanding of tinnitus mechanism. The results also highlight potential neural targets for neuromodulation interventions addressing both sensory and cognitive components of chronic phantom perception.

## 1 Introduction

Auditory phantom perception is a perceptual experience without a corresponding stimulus in the external world (Smith, 1861). This is the characteristic hallmark phenotype for tinnitus, the continuous ringing of the ear in the absence of an external sound source (Baguley et al., 2013). It is one of the most common complaints in patients with hearing impairments (Bhatt et al., 2016;Hackenberg et al., 2023), with tinnitus being 7.7 times more likely than in those who do not have hearing loss (Biswas et al., 2023). This suggests an influential contribution of bottom-up sensory deficits in generating auditory phantom perception. Alternatively, auditory phantom perception may also occur in patients with undetectable sensory loss, such as tinnitus in patients with “hidden hearing loss” (Schaette & McAlpine, 2011). This emphasizes the top-down modulation in the mechanism of auditory phantom perception (De Ridder et al., 2014;Hullfish et al., 2019).

Recent research has demonstrated distinct neural networks involved in phantom perception triggered by bottom-up and top-down factors (Aleman & Vercammen, 2013;Hong et al., 2016;Vanneste et al., 2019). Yet, in a clinical population like tinnitus, it is challenging to rule out the effects of distress or co-morbid mental disorders such as anxiety and depression to study the influence of bottom-up and top-down modulatory factors on the perceptual component itself (Bhatt et al., 2016;Jarach et al., 2022). Alternatively, auditory illusions—experimentally induced phantom perception—could serve as physiological and causal models to explore the neural processing and networks driven by different factors. For instance, by manipulating auditory stimuli, researchers are able to elicit numerous auditory illusions, such as auditory continuity illusion (Miller & Licklider, 1950), the Precedence Effect (Wallach et al., 1949;Wallach et al., 1949), and Zwicker Tone illusion (Zwicker, 1964). In contrast to bottom-up factors, auditory illusions can also be driven by multisensory conflicting information, such as McGurk effect (McGurk & MacDonald, 1976) or strong expectations to multisensory association, such as the Conditioned Hallucination (Ellson, 1941). The current study, therefore, aims to use two types of auditory illusions as human models/proxies for tinnitus to dissect the neural correlates of bottom-up and top-down factors in the purely perceptual processes, leading to insights into the underlying neural mechanism of phantom auditory perception. Amid various auditory illusions, we focused on the Zwicker tone (ZT) and Conditioned Hallucinations (CH) due to their perceptual similarity to tinnitus, as both are experienced as simple tones—a common characteristic of tinnitus perception. Moreover, these two auditory illusions are driven by distinct mechanisms: ZT primarily reflects bottom-up sensory processes, while CH is influenced by top-down cognitive factors.

The effect of*bottom-up*factors on the occurrence of tinnitus is well known. Yet, its underlying mechanism and neural network remain debatable (Aleman & Vercammen, 2013;Marschall et al., 2020). “Sensory-like” experiences could result from lesions or deafferentation in the auditory system that leads to disinhibition in the central nervous system (Noreña & Farley, 2013). The intrinsic excitability and the interference with inhibitory networks of the sensory system are enhanced to restore homeostasis (Marschall et al., 2020). Tinnitus is then hypothesized as the perception of increased noise in the auditory system, driven by the increased central “gain” or disinhibition through networks connecting the sensory cortex with other cortical regions (De Ridder et al., 2014;Noreña, 2011). From a predictive coding perspective, this disinhibition and increased excitation are considered to increase the strength of the evidence for perceiving a phantom which, over a certain point, overrides the brain’s prediction to perceive silence in the absence of a stimulus to actively perceive tinnitus (Sedley et al., 2016).

A scenario of deprived sensory input can be experimentally modelled by exposing participants to a notched noise (broadband noise with a filtered frequency band). This induces a temporary and auditory illusion which is an afterimage of the missing central frequency called the Zwicker Tone (ZT) (Zwicker, 1964), with the perception rate ranging from 47% to 90% across studies (Mohan et al., 2020;Zwicker, 1964). Previous studies find that the width of 0.75–1 octave and central frequency >4000 Hz are essential characteristics for the success of the afterimage (Leske et al., 2014;Mohan et al., 2020;Noreña & Eggermont, 2003). Given these characteristics, the ZT illusion has been hypothesized to be a “reversible” model of tinnitus driven by hearing loss (Noreña & Eggermont, 2003;Norena et al., 2000). In tinnitus patients with hearing loss, the pitch of tinnitus is usually located at or around the edge of the hearing loss frequency (Nicolas-Puel et al., 2002), which is most common in higher frequencies.

Recent studies proposed a remodelling of lateral inhibition along the auditory pathway as the potential neural mechanism of the ZT. Lateral inhibition among neurons in the central auditory system (e.g., the dorsal cochlear nucleus) enhances the spectral contrast of sensory inputs by emphasizing differences in activity between neighboring units on topographical maps (Norena et al., 2000;Pantev et al., 1999;Rhode & Greenberg, 1994). During the ZT illusion, neurons within the notch band receive little or no excitation from the stimulus but are subsequently disinhibited (Franosch et al., 2003). The consequently increased activity response to the centre of the spectral gap is perceived as the illusion (Franosch et al., 2003). A similar neural pathophysiological process was also observed in tinnitus after sensory loss (Gerken, 1996;Pantev et al., 2012). Alternatively, more recent evidence demonstrated the role of neural oscillations, especially the alpha oscillation, in the modulation of the ZT illusion discrepancies between inhibitory and excitatory processes in the auditory cortex (Leske et al., 2014), as well as involvement of high-order regions, such as parahippocampus and cingulate cortex (Mohan et al., 2020) that are part of the “noise‐cancelation” sensory gating system (Rauschecker et al., 2010). Overall, the close links between the ZT and tinnitus were evident at both phenomenological and neurophysiological levels.

In contrast, the role of*top-down*factors in tinnitus has also been widely studied (Aleman & Vercammen, 2013;Hullfish et al., 2019;Vanneste et al., 2019). Neuromodulation studies show the involvement of higher order non-auditory regions such as the pregenual anterior cingulate cortex/ventromedial prefrontal cortex (pgACC/vmPFC), dorsal anterior cingulate cortex (dACC), and parahippocampus to modulate the intensity and severity of the tinnitus (De Ridder & Vanneste, 2014;Kreuzer et al., 2015;Vanneste & De Ridder, 2011). The pgACC/vmPFC has been hypothesized to be part of a top-down “noise cancellation” system that possibly controls if the “noise” generated due to deafferentation at the level of the thalamus reaches the cortex (Mühlau et al., 2006;Rauschecker et al., 2010). The dACC, part of the salience network, is a core region that controls tinnitus severity (De Ridder et al., 2022;Shin et al., 2011). Additionally, dACC is significantly more activated in people with post-traumatic stress than their monozygotic twin who was not exposed to trauma (Shin et al., 2011). Furthermore, stress-related exhaustion decreases pre-frontal cortex and anterior cingulate volume (Grossi et al., 2015). Stress is a major risk factor for tinnitus, which modulates its intensity through the connection between the anterior cingulate and auditory regions (Vanneste et al., 2014). Finally, the auditory memory plays an important role in the generation of tinnitus (De Ridder et al., 2011). From a predictive coding perceptive, it is hypothesized that tinnitus may be generated due to a system that is unable to adapt to the changing environmental needs, possibly owing to an overly precise internal model (Yasoda-Mohan et al., 2024). In other words, if the brain is unable to adapt to the hearing loss, to change its expectation of the world to*not*expect the frequencies that are lost, the brain would have to compensate for this unchanging expectation by putting out a phantom sound. This is called the strong prior hypothesis (Corlett et al., 2019).

This scenario can be modelled using an auditory illusion called Conditioned Hallucination (CH) (Ellson, 1941;Powers et al., 2017). In this paradigm, an association between an uncertain auditory target and a visual cue is first learned. When only the cue is played in the absence of the tone, it elicits an illusion of the tone in those who built a stronger internal model of the learned association (Powers et al., 2017). Such an illusory experience is a cued response that aligns with the concept of overweighed perceptual expectations (Powers et al., 2017). More specifically, hallucinators showed greater activation in the superior temporal sulcus and anterior insula cortex, which were associated with auditory expectations and multisensory integration (Powers et al., 2017). The concurrent computational modelling revealed that hallucinators weighed more on prior expectations than sensory evidence, indicating that visual cues elicited stronger auditory expectations that eventually contribute to CH. Notably, the CH illusion has been employed to investigate the mechanism of various perceptual disorders by examining the interplay of prior expectations and sensory evidence in perception. For instance, the fluctuation in the CH rate aligns with changes in the frequency and intensity of hallucinatory experiences over time (Kafadar et al., 2022). Similar to individuals with hallucinations, those with tinnitus demonstrated a higher propensity for experiencing the CH illusion (Yasoda-Mohan et al., 2024). This heightened susceptibility to CH illusion is thought to stem from the tendency of tinnitus patients to construct stronger internal models based on multisensory associations, leading them to automatically compensate with the missing information based on their model. Thus, all these findings suggest a shared or overlapped top-down neural mechanism between tinnitus and the CH illusion.

While previous research has independently explored these illusions, this study leverages a within-subject design to understand the neural mechanisms underlying ZT and CH illusions. By inducing the two illusions in the same healthy sample, we can analyse bottom-up and top-down factors within each condition separately to understand how they contribute to causal perceptual inference. This approach may resolve the challenge of studying these effects cross-sectionally in clinical models, which would need copious resources for longitudinal studies and risk confounding influences from tinnitus comorbidities and pathophysiological mechanisms (e.g., distress). The current study also uses Granger causality (GC) analysis to directly compare causal connectivity patterns between the ZT and CH conditions, investigating distinct and overlapping neural pathways involved in auditory perception. This design offers novel insights into the mechanisms of perception without relying on complex clinical models.

Thus, this study aimed to model the neurophysiological signatures and networks of two phantom perceptions induced by modulating either bottom-up or top-down factors. We hypothesized that (1) both ZT and CH would induce different neural responses that coincide with illusory experiences, (2) more bottom-up processes would be involved in the perception of ZT and more top-down process would be involved in the perception of CH, and (3) these results will help us deepen our understanding of tinnitus. By investigating diverse auditory illusion models, we not only explore the neural mechanisms underlying these processes but also put forward their great potentials for tinnitus research, providing a deeper understanding of the perceptual and cognitive dimensions of tinnitus. Importantly, the current research underscores the role of non-auditory cognitive systems in shaping auditory phantom perception, offering novel insights into potential neural markers and target regions for neuromodulation. Furthermore, this study quantitatively examines how different auditory illusions relate to neural mechanisms that may be relevant for tinnitus research, contributing to the bridge between experimental models and clinical phenomena. These findings could also have significant implications for developing therapeutic approaches that address both sensory and cognitive components of tinnitus.

## 2 Materials and Methods

### 2.1 Participants

All participants gave their written, informed consent per the EU General Data Protection Regulation 2016 (GDPR). Participants were also screened for medical history using an online screening questionnaire. Exclusion criteria consist of continuous phantom perception (tinnitus, verbal hallucination, phantom pain), chronic ear disorders (e.g., Meniere’s disease, ear infections, otosclerosis), and several neurological disorders such as tumours, mental health disorders, and chronic headaches. In addition, all participants completed a set of questionnaires including Beck’s Depression Inventory (BDI), Beck’s Anxiety Inventory and Predisposition of Hallucination Scale, which were applied to screen for mental health and assess the predisposition to hallucinations in healthy individuals. Participants who scored BDI higher than 13 were excluded. Meanwhile, they also underwent an online pure tone test where bilateral hearing thresholds at 500, 1000, 2000, 3000, 4000, 6000, and 8000 Hz were obtained. Those with hearing thresholds at any frequency >30 dB HL were excluded from the study.

To ensure the study’s statistical rigor, we performed power calculations using an independent samples t-test. The alpha level was set at 0.05, with a statistical power of 0.8. Based on the effect size (Cohen’s d = 1.0) determined by previous work in our laboratory (Mohan et al., 2020), the assumed total sample size required to achieve the desired significance level was 46. Forty-seven healthy young adults were screened for inclusion criteria. Per the exclusion criteria, 2 out of 47 participants were excluded due to mild-to-moderate hearing loss (n = 1) or mild mood disturbance reflected by Beck’s Depression Inventory (BDI) (n = 1). Among 45 participants, 3 dropped out after one session, 3 did not perform correctly during experiments (fell asleep or repeated one response throughout the response-required experiments), and 2 reported unreliable responses in the Zwicker Tone session (explained below), leaving 37 participants.

**Fig. 1.**
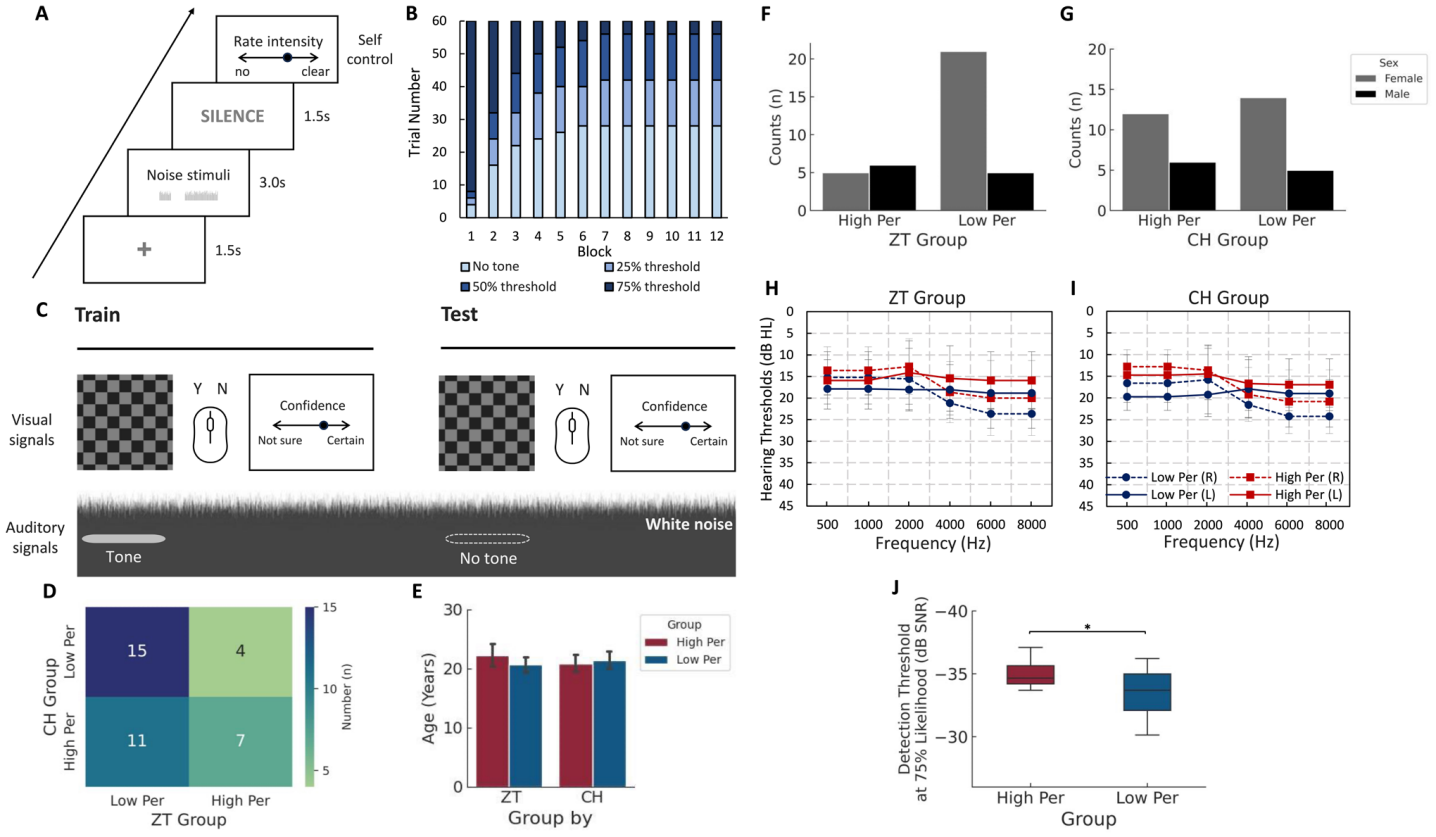
Trial structures of two illusion paradigms and demographical plot. (A) Zwicker Tone (ZT) paradigm. Noise stimuli in the ZT paradigm consisted of 4k notched noise, 1k notched noise, and white noise. The sequence of stimuli was pseudo-randomized by presenting the three different stimuli in a random order. During the experiment, participants were asked to direct their gaze at the fixation cross followed by a noise stimulus for 3 s. Participants were then required to rate the clearness of a ringing sound they may perceive during the silent time, via a visual analogue scale (VAS). (B) Trial number distributions for the Conditioned Hallucination (CH) paradigm. Four stimulation conditions were estimated individually based on the psychometric curves of the tone detection from the QUEST test and the proportion of four conditions was structured in a non-linear manner. Threshold tones were more likely early and absent tones were more likely later, with systematically varied steps over 12 blocks of 60 conditioning trials. (C) Trials in the CH paradigm consisted of a visual checkerboard and a simultaneous 1k Hz tone with a specific stimulation condition. During the experiment, participants were required to indicate whether they heard an auditory tone when a visual checkerboard was present by clicking the mouse. Participants were then asked to rate their confidence in this decision via a VAS from “Not sure” to “Very certain.” (D) The number of high and low perceivers for ZT and CH conditions. (E) Age comparison between high and lower perceivers in ZT and CH sessions. Sex distribution between high and low perceivers in ZT (F) and CH session (G). Hearing thresholds for high and low perceivers in ZT (H) and CH session (I). (J) Detection thresholds at 75% tone threshold between the CH high and low perceiver groups. Data are present with mean with the error bar of 95% CI.*p<.05.

All 37 participants were divided into high perceiver or lower perceiver groups based on their behavioural responses in each illusion condition. Based on the ZT session, 11 participants were classified as ZT high perceivers, whereas 26 were classified as ZT low perceivers. For the CH session, 18 were classified as CH high perceivers and the other 19 were classified as CH low perceivers. More details are described below in the*Behavioural data analysis*section. There was no significant difference in the number of high and low perceivers between the two sessions (*X^2^(1)=0.683, p*= .408), suggesting a limited association between the susceptibility of two auditory illusions (Fig. 1D). The age (ZT:*t(35)=1.377, p=.184*; CH:*t(35)=0.490, p=.627*) (Fig. 1E), sex (ZT:*X^2^(1)=3.079, p=.079*; CH:*X^2^(1)=0.011, p=.915*) (Fig. 1F, G), and hearing status (ZT:*F(3,33)=0.180, p=.910, η²_p_=.016*; CH:*F(3,33)=1.184, p=.164, η²_p_=.142*) (Fig. 1H, I) were not significantly different between high and low perceivers for the ZT and CH sessions.

### 2.2 Experiment summary

All participants underwent the ZT and the CH paradigm in 2 consecutive days, with an additional behavioural session for the CH paradigm to estimate individual psychoacoustic thresholds. The sequence of the two paradigms was fully randomized among participants to rule out any order effect. Each session included 30-min preparation for EEG recording and 45-min auditory illusion paradigm.

### 2.3 Behavioural session

The determination of the detection threshold for each participant was accomplished via the QUEST maximum likelihood-based procedure through MATLAB Psychtoolbox 3.0 (Watson & Pelli, 1983). The QUEST procedure consisted of 2 runs of 40 trials with initially ascending interleaved staircases of step size based on the participant responses. Participants were required to indicate the presence of 1k Hz pure tones embedded in 40 dB SPL noise, which was concurrent with a visual checkerboard cue. The checkerboard was a grey and black checkerboard with grey squares at 25% brightness. Each trial in the behavioural part consisted of the 300 ms audio-visual (target-cue) stimulus embedded in continuous background noise and 500–1000 ms inter-trial interval. Participants were asked to indicate the presence or absence of the auditory stimulus concurrent with the visual cue as soon as possible using the left/right mouse click. The left/right click indicating “yes”/“no” was also randomized across subjects. The procedure ended with the estimation of the 75% likelihood detection threshold of the auditory tone. The 50% and 25% likelihood detection thresholds for each subject were further calculated using the psychometric curves. The averaged 75%, 50%, and 25% likelihood detection thresholds of auditory stimuli across the two runs were used in the electrophysiological session of the CH paradigm. There was a significant difference in the 75% likelihood detection threshold between CH high and CH low perceivers (Fig. 1J).

### 2.4 Electrophysiological session

The ZT paradigm and CH paradigm were applied to induce two types of auditory illusions. All audio files and visual stimuli used in the two paradigms were produced in MATLAB.

#### 2.4.1 ZT paradigm

The stimuli consisted of 3-s-long white noise (WN), notched noise with a central frequency of 4000 Hz (4k-NN) as the most sensitive frequency, and notched noise with a central frequency of 1000 Hz (1k-NN) as the least sensitive frequency to produce a ZT illusion. The selection of two frequencies was based on the previous studies (Leske et al., 2014;Mohan et al., 2020). The bandwidth of notch noise was 1-octave width as the intensity rating of ZT is maximal at that width (Leske et al., 2014). The stimuli were played binaurally at 60 dB SPL. All audio files were matched for overall RMS power. Each trial started with a 1.5 s fixation cross, which was followed by a noise stimulus for 3 s. After a 1.5 s silent period, participants were required to rate the clearness of a ringing sound they may perceive during the silent time, via a visual analogue scale (VAS). This VAS used a 6 cm line with end point descriptors as “No” at the left end and “Very clear” at the right end (Fig. 1A). The paradigm consisted of 3 blocks of 100 trials. The sequence of three stimuli was pseudo-randomized in a series of three to avoid the suppressed perception due to the repetitive exposure of the same stimulus.

#### 2.4.2 CH paradigm

The target auditory stimulus consisted of a 300 ms 1k Hz pure tone with a rise–fall time of 10 ms embedded in 40 dB SPL background noise. The sound levels of the stimulus correspond to 75%, 50%, and 25% likelihood of detection thresholds in addition to tone-absent conditions. The proportion of four stimulus conditions was structured in a non-linear manner, such that a disproportionately larger number of threshold-level (75% and 50%) stimuli were presented in the initial runs, followed by an overrepresentation of sub-threshold (25%) and absent stimuli in the later runs (Fig. 1B). A 4 x 7 checkerboard with grey and black squares served as a visual cue. The checkerboard was presented at 25% brightness to maximize visual stimulation while minimizing lasting after effects. Each trial started with a 300 ms visual stimulus concurrent with an auditory stimulus if present, followed by a 1000 ms period to develop induced activities. Participants were then required to indicate whether they heard a tone along with the checkerboard within a 1500 ms response window with a mouse click. The left or right mouse click was pseudorandomized to ensure equal numbers. Participants were then asked to rate their confidence in this decision within 2000 ms, via a 4 cm VAS from “Not sure” to “Very certain” (Fig. 1C). The inter-trial interval was jittered between 300 and 500 ms. Those trials in which participants failed to indicate the presence of auditory sound within 1500 ms or to rate their confidence within 2000 ms were marked as miss and excluded from ERP pre-processing. The paradigm consisted of 4 functions run in which 3 blocks of 60 trials were included. Participants were implicitly taught the association between two stimuli in the beginning blocks and then tested for the perception of CH in the latter blocks. The block was performed sequentially, whereas the sequence of trials in each block was fully randomised.

### 2.5 Behavioural data analysis

#### 2.5.1 ZT session

All participants were screened for their ability to reliably perceive the ZT based on the same criterion used in the previous studies (Mohan et al., 2020;Qi et al., 2022). This was defined as rating 2 or less to the WN and 1k NN in less than 50% of total trials, respectively. The participants who did not satisfy this criterion were excluded from further analysis due to their unreliable responses to the stimuli. Then, ZT high perceivers were defined as rating 2 or more to the 4k NN in more than 50% of total trials. Based on the criteria, 11 participants were classified as ZT high perceivers whereas 26 were classified as ZT low perceivers. Subjects’ positive response rate for all three conditions was calculated by the ratio of the number of trials where they rated 2 or more and the total number of trials (n = 100) within a condition. The mean intensity of the ZT illusion within each stimulation condition was calculated per subject. Repeated measures ANOVA was performed to analyse the difference in positive response rate and mean intensity by using the ZT condition as a within-subject factor and the ZT group as a between-subject factor. Due to the significant smaller number of participants in the ZT high perceiver group, the non-parametric test—Mann–Whitey U test was applied for the post hoc pairwise comparison between the two groups and corrected for multiple comparisons by the Benjamini Hochberg correction (FDR rate of 5%).

#### 2.5.2 CH session

All subjects were identified as 18 high CH perceivers or 19 CH low perceivers based on the median FA rate. Then, the CH group difference in the response rate and confidence rating was analysed. The average positive response rate at each tone threshold condition was calculated as the ratio of the number of trials saying “Yes” and the total number of trials in that condition after controlling for the number of trials with no response. The positive response numbers at the 75% and 0% threshold conditions over the blocks were calculated per subject and checked to rule out the possibility of response bias. The average confidence rating in positive (saying “Yes”) and negative (saying “No”) responses at the 0% threshold was calculated over the blocks in each tone threshold. Repeated measures ANOVA was performed to analyse the difference in the positive response rate as well as confidence rating with the between-subject factor as CH group. The within-subject factor for the analysis of positive response rate was the threshold condition, whereas the within-subject factors for the analysis of confidence rating were the threshold condition and response. Post hoc pairwise comparisons between the two groups were conducted by using the independent samples t-test and corrected for multiple comparisons by the Benjamini Hochberg correction (FDR rate of 5%). A linear mixed-effect model (LME) was applied where the positive response number at individual thresholds or confidence rating at 0% threshold were the independent variables. Subjects were set as the random factor. The fixed factors for the LME of response number were group and block, while the fixed factors for the LME of confidence rating were group, block, and response. Post hoc pairwise comparisons between the two groups were conducted by using independent samples t-test and corrected for multiple comparisons by the Benjamini Hochberg correction (FDR rate of 5%).

### 2.6 EEG data collection and pre-processing

The stimuli in the electrophysiological session were presented through Psychopy which triggered the Biosemi ActiView software. The ERP data were collected using a 64-channel cap configured as per the International 10–20 placement system. The data were sampled at 4096 Hz and recorded by the BioSemi ActiveTwo system. Data were pre-processed using MATLAB, EEGLAB v2021.1, and ERPLAB v8.20. The pre-processing pipeline included removing disconnected and unused channels, decreasing the sampling rate to 500 Hz, re-referencing to an average reference, and filtering between .55 and .44 Hz. The high-pass filter of .55 Hz was chosen to minimize low-frequency artefacts while providing a better quality of EEG activity (Delorme, 2023). The data were epoched based on the trial structure of individual paradigms. For the ZT paradigm, the epoch was defined as -600 to +4500 ms relative to the onset of the noise stimulus. For the CH paradigm, the epoch was defined as -300 to +1300 ms relative to the onset of the target stimulus. The epoched data with all conditions were then subjected to temporal independent component analysis (ICA) to remove muscle artefacts, eye blinks, saccades, and other noise transients. Artefacts in all epochs were detected and deleted using a simple voltage threshold of ±90 µV and manual inspection. The channels initially removed were finally interpolated using a spherical interpolation algorithm in EEGLAB. All epoched data were baseline corrected based on the pre-stimulus activity.

### 2.7 Event-related potential (ERP) analysis

To specify the neural correlates to each auditory illusion, the neural responses for the two types of illusions were investigated. For the ZT session, all trials in the 4k-NN condition were divided into the ZT trial condition and no ZT response (NR) trial condition. The trial numbers for each subject used for the comparison are listed inSupplementary Table S1. Then, ZT trials were averaged per subject in the ZT high perceiver group and so as for the NR trials in the ZT low perceiver group. Average ERPs were compared between the two groups using cluster-based permutation tests. For the non-parametric cluster-based permutation test, an independent samples t-test was calculated for each channel-time sample between the two groups with the alpha of*.05*as the cluster-building threshold (two-tailed) (Maris & Oostenveld, 2007). The neighbour channels were calculated using a standard template of the Biosemi 64 channel cap provided from the Field trip. The minimum number of neighbouring channels to form a cluster was 2. The resulting clusters were corrected for multiple comparisons using max-T, which takes the sum of the t-values within every cluster and compares them with the probability of clusters that were calculated by Monte-Carlo of 5000 permutations (Maris & Oostenveld, 2007). Clusters that were larger than 95% of the distribution of clusters were considered significant (*α=.05*, two-tailed). The clusters that showed significant group differences were selected.

For the CH session, all trials in the absent condition were divided into false alarms (FA) or correct rejections (CR). Then, FA trials were averaged per subject in the CH high perceiver group and so as for the CR trials in the CH low perceiver group. Averaged ERPs were compared between the two groups by using the cluster-based permutation independent samples t-tests, following the same procedure as described above.

Given the significantly smaller number of trials in the FA condition compared with the CR condition (Supplementary Table S1), we addressed this imbalance by randomly subsampling 50 trials (the average number of FA trials) from the CR condition for each subject in the low perceivers group. The trial-averaged CR ERPs from the low perceivers were then compared with the trial-averaged FA ERPs from the high perceivers. This process was repeated 100 times to generate a t-test map. Electrodes showing significant results in at least 80 out of 100 comparisons were selected for plotting.

For the ERP plot, we plotted the ERP that showed significant group difference. In this way, the baseline time of ERP plot for ZT session was the offset of notched noise (i.e., onset of silence where participants perceived ZT), whereas the baseline time of ERP plot for CH session was the onset of absent tone trial (i.e., onset of the visual checkerboard).

Based on the cluster-based permutation test, the mean amplitudes of ERPs were calculated by averaging across significant time-channel clusters (seeFig. 3A, D) for each illusion session and subject. Then, the Pearson correlation coefficient test was applied to measure the correlation between the evoked neural responses and behavioural responses (*α=.05*, two-tailed). For the ZT session, the mean amplitudes of 4k-NN evoked ERPs were correlated with the percentage of ZT perception and the subjective intensity scores of ZT perception. For the CH session, the mean amplitudes of absent tone evoked ERPs were correlated with the percentage of CH (i.e., FA) and subjective confidence in reporting CH. To control for the multiple comparisons, the Benjamini Hochberg correction was applied with the FDR rate of 5%.

### 2.8 Time–frequency analysis

To further delve into the neural correlates of the two auditory illusions, time–frequency decomposition was performed for each group in two sessions (i.e., ZT, NR, FA, and CR). This was done by convolving a family of Morlet wavelets with a logarithmically increasing number of cycles between 3 cycles at 4 Hz and 12 cycles at 43 Hz. The time–frequency decomposition was analysed per single trial per group for all channels. Baseline normalization of total power was then applied by using the decibel conversion (-350 to -50 ms for the ZT session and -300 to 0 ms for the CH session). Given the limited pre-stimulus time window for both session, 5100 ms (i.e., the time length for the whole trial) of ZT reflected data was concatenated to the beginning and end of the real signal before the single-trial data were first decomposed to minimize potential edge artefacts and subsequently discarded. For the CH session, 1600 ms of reflected data was concatenated to the beginning and end of the real signal before the single-trial data were first decomposed to minimize potential edge artefacts and subsequently discarded.

Then, the statistical difference of the time–frequency data was computed by the cluster-based permutation test between ZT of high perceivers and NR of low perceivers and between FA of CH high perceivers and CR of low perceivers (Maris & Oostenveld, 2007). The key parameters were same as those for the cluster-based permutation test for the ERP data. Considering the enormous computing power for the time–frequency cluster-based permutation test, the analysis was performed for individual frequency bands. Four frequency bands were defined as theta (4–7.5 Hz), alpha (8–12 Hz), beta (13–30 Hz), and gamma (30.5–43 Hz).

Third, the average total power was calculated by averaging across channels that showed significant group differences in time–frequency samples (seeFig. 3G, K). The difference in the average total power was compared by the independent samples t-test between the two groups in each session (*α=.05*, two-tailed). Then, the Pearson correlation coefficient test was applied to measure the correlation between the average total power and behavioural responses (*α=.05*, two-tailed). For the ZT session, the total powers were correlated with the percentage of ZT perception and the subjective intensity scores of ZT perception. For the CH session, the total powers were correlated with the percentage of CH and subjective confidence in reporting CH. To control for the multiple comparisons, the Benjamini Hochberg correction was applied with the FDR rate of 5%.

### 2.9 Source localization

Subject-average ERPs to individual trial conditions were source localized using Brainstorm toolbox (Tadel et al., 2011). The ERPs were averaged across 600 to 900 ms after the offset of the noise for ZT and NR conditions and 276 to 392 ms post-stimulus for FA and CR conditions based on the ERP analysis. ICBM-1522 template was selected for the head model and the forward model OpenMEEG BEM. The noise covariance matrix was calculated based on the 100 ms baseline before the first stimulus. Source reconstruction was performed using dynamic statistical parametric mapping (dSPM). The baseline normalization was conducted by z-score transformation (-550 to -50 ms for the ZT session and -300 to 0 ms for the CH session).

To specify the regions that were involved in the perception of two auditory illusions, source signals were averaged within regions based on the Desikan–Killiany Atlas per trial (Desikan et al., 2006) and were compared between the two groups in each session by the region-based permutation test. The null distribution of the group difference was calculated by the permutation of independent samples t-test for 5000 iterations. Considering the potentially spurious results and its impact on the following analysis, we conducted more conservative statistical thresholds: only the observed difference (t values) that was larger than 97.5% of the distribution was considered significant (*α=.025*, two-tailed).

### 2.10 ROI identification

Region of interest (ROI) selection for connectivity analysis is critical, as the computational complexity of the Granger causality analysis increases quickly when the number of ROIs increases. Including too many ROIs might consequently result in overfitting models and undermining the sensitivity of models (Gow & Caplan, 2012). In this way, we conducted a more conservative statistical threshold for the source localization analysis as explained above. To find the connectivity pattern for each auditory illusion, those regions that showed significant group differences in each auditory illusion session were selected as the ROIs for the connectivity analysis, respectively. Furthermore, to find differences in the patterns of connectivity between the two auditory illusions, we only included the significant GC in either session, in order to prevent overfitting of the GC computation.

### 2.11 Granger causality

To further investigate the neural networks of two auditory illusions and differentiate them from each other, the Granger causality (GC) was applied. GC measures the predictability of the signal from one region to another over the time series, which is quantified as the strength of directed connectivity. GC is constructed based on linear vector autoregressive (VAR) models, in which the signals in later time points are predicted by a linear combination of previous samples. If previous signals contribute to prediction models reflected by the reduced variances of modelling errors, one time series is believed to Granger cause a second one, with a higher value implying higher Granger causal. The implementation of Granger causality requires two main assumptions: (1) GC assumes that the time series have random variations and can be modelled as stochastic processes. This is essential because GC relies on comparing model prediction errors. (2) GC typically requires that the data are stationary, meaning the probability distribution of each series remains constant over time (Seth et al., 2015). This ensures consistency in the statistical properties of the time series.

Here, we conducted the GC analysis to explore the underlying linear relationship among the ROIs identified in the source localization section for each auditory illusion. Considering that ERP data are non-stationary, three preprocessing steps were conducted for all time series data in all subjects, including detrending to remove drifts and slow fluctuations, demeaning to remove ERP components and differencing to remove non-stationary components (Seth et al., 2015). To verify the stationarity, pre-processed data were evaluated by the KPSS test to check for unit roots (Kwiatkowski et al., 1992). The results showed that trial-based non-stationary rate for each subject and each session was below 1.7% (ZT: 0.32% ± 0.41%, CH: 1.68% ± 2.54%). Then, model order, which is the maximum number of lagged observations, was determined by the Bayesian Information Criterion (BIC) test (Schwarz, 1978). The range of model orders set as 1 to 20. The optimal model order is that resulted in the minimum value of BIC test. Based on this criterion, the optimal model order was 10 for the ZT session, 6 for the CH session, and 8 (averaged by 2 optimal model orders) for the comparison between the 2 illusions. The scout function was aggregated before the measure and time windows were determined as the significant time series from individual ERP analysis: post-stimulus 600 to 900 ms for the ZT session and post-stimulus 276 to 392 ms for the CH session.

The GC analysis was first analysed for each session to capture the network patterns of each type of auditory illusion. For the ZT session, the six ROIs that showed significant group difference at the source localization analysis were included. The GC of all ROIs was calculated for each ZT trial of the ZT high perceivers and each NR trial of the ZT low perceivers. For the CH session, the ROIs were selected also based on the source localization results. The GC of all ROIs was calculated for each FA trial of the CH high perceivers and each CR trial of the CH low perceivers. Then, for each condition, the connectivity matrix was averaged over trials for each ROI in each subject. In each session, the averaged connectivity matrix was further compared between the two perceiver groups by the permutation test. The null distribution of the group difference was calculated by the permutation test with 5000 times of randomization. Only the observed difference that was larger than 95% of the null distribution of difference was considered significant (*α=.05*, two-tailed). To control for the multiple comparisons, the Benjamini Hochberg correction was applied with the FDR rate of 5%.

Furthermore, we directly compared the difference in connectivity patterns between the two auditory illusions. First, only the significant GC in either session was selected for the following analysis to prevent overfitting of the GC computation. In this way, 22 significant GC connections were included. Then, the GC was calculated for each ZT trial of the ZT high perceivers and each FA trial of the CH high perceivers. To compare the connectivity networks of two illusion models, the permutation test was performed with the same procedure as mentioned above. To control for the multiple comparisons, the Benjamini Hochberg correction was applied with the FDR rate of 5%.

## 3 Results

### 3.1 Behavioural results

#### 3.1.1 ZT session

**Fig. 2.**
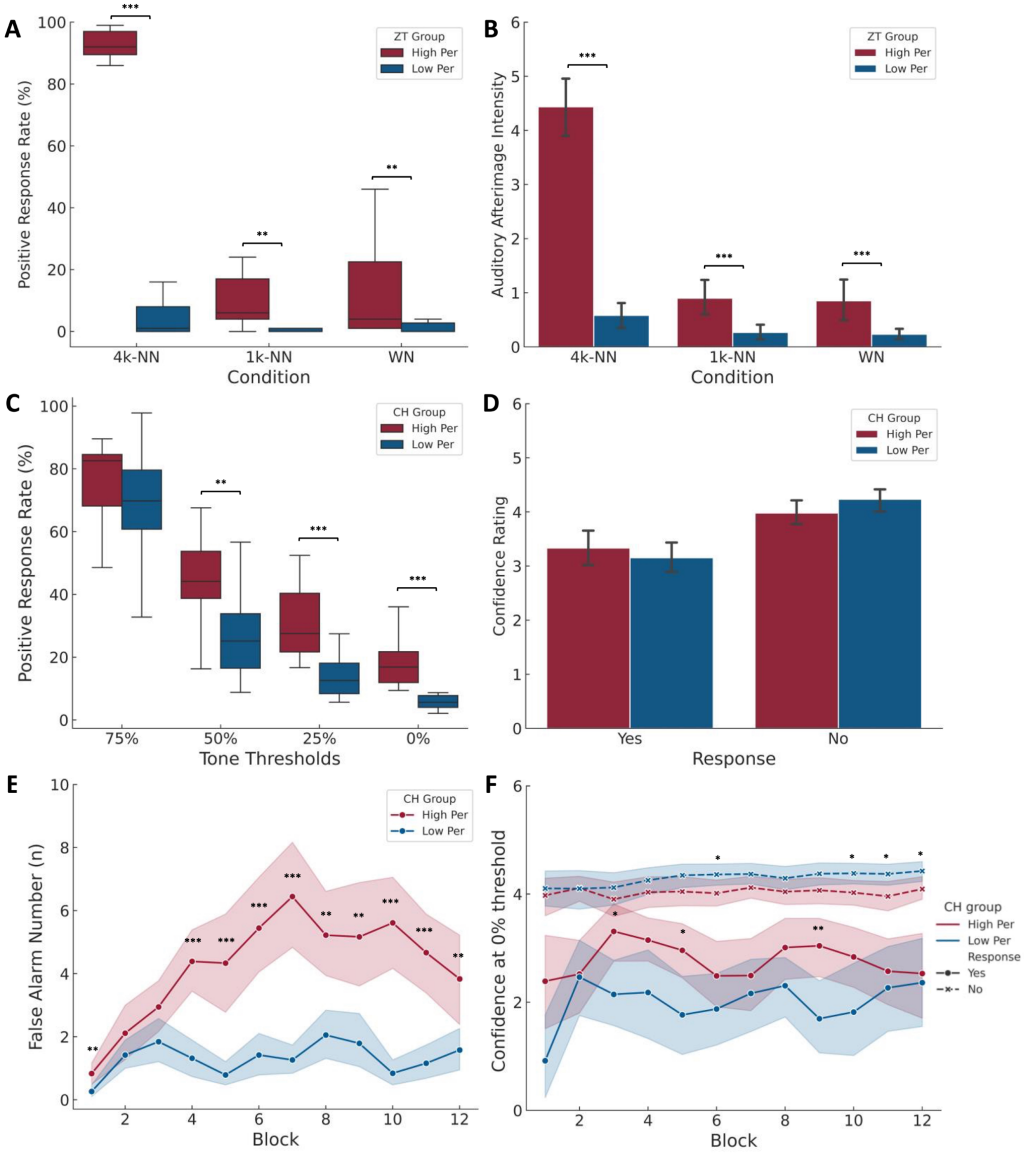
Behavioural data for the ZT and CH session. (A) Group difference of the positive response rate between the ZT high and low perceiver groups in the 4k-notch noise (NN), 1k-NN, and white noise (WN) conditions. (B) Group difference of the mean ZT illusion intensity between the two ZT groups in the 4k-NN, 1k-NN, and WN conditions. (C) Difference of the mean positive response rate between the CH high and low perceiver groups in four tone threshold conditions. (D) Group difference of the mean confidence rating in positive and negative responses between two CH groups. (E) Group difference of the false alarm number in the 0% threshold condition over the blocks between two CH groups. (F) Group difference of the confidence rating in positive and negative responses in four tone threshold conditions over the blocks between two CH groups. Data were present with mean with the error bar of 95% CI.*p<.05 **p<.01 *** p<.001.

We first analysed the difference in the behavioural responses to ZT perception between the two ZT groups, including positive response rate and mean ZT intensity. For the response rates, we observed a main noise condition effect (*F(2,70)=256.477, p<.001, η²_p_=.880*), a main ZT group effect (*F(1,35)=119.715, p<.001, η²_p_=.774*), and an interaction effect of noise condition*ZT group (*F(2,70)=193.439, p<.001, η²_p_=.847*) (Fig. 2A). Compared with the ZT low perceivers, the ZT high perceivers showed a significant increase in the response rates for all three noise conditions (4k-NN:*z=4.839, p<.001*; 1k-NN:*z=3.097, p=.004*; WN:*z=3.293, p=.001*). For the mean intensity, the results also showed a main noise condition effect (*F(2,70)=187.568, p<.001, η²_p_=.843*), a main ZT group effect (*F(1,35)=137.180, p<.001, η²_p_=.797*), and an interaction effect of noise condition*ZT group (*F(2,70)=129.015, p<.001, η²_p_=.787*) (Fig. 2B). The ZT high perceivers also rated significantly higher intensity of illusions in all three noise conditions than the ZT low perceivers (4k-NN:*z=4.752, p<.001*; 1k-NN:*z=3.555, p<.001*; WN:*z=3.389, p<.001*). These results are in line with previous studies from our and other laboratories (Leske et al., 2014;Mohan et al., 2020;Norena et al., 2000).

#### 3.1.2 CH session

Furthermore, we analysed the difference in the behavioural responses in the CH paradigm between the two CH groups, including the average positive response rates and average confidence rating for each threshold condition. The repeated measures ANOVA revealed that there was a main threshold condition effect (*F(3,105)=314.727, p<.001, η²_p_=.900*) and a main CH group effect (*F(1,35)=19.734, p<.001, η²_p_=.361*), but no significant interaction effect of threshold condition*CH group on the positive response rate (*F(3,105)=2.490, p=.064, η²_p_=.066*). The post hoc pairwise comparisons showed no group difference in the positive response rate in the 75% tone threshold (*t(35)=1.305, p=.200*), but significantly higher response rates of the CH high perceivers during the 50% (*t(35)=2.991, p=.005*), 25% (*t(35)=5.566, p<.001*), and 0% (*t(35)=7.178, p<.001*) tone thresholds (Fig. 2C). These post hoc differences suggest that the interaction effect may be approaching statistical significance rather than being entirely absent, reflecting underlying trends in the data. This is consistent with the hypothesis that CH high perceivers are more prone to build strong priors that would bias the perception as the sensory environment becomes more uncertain.

Furthermore, the confidence in positive and negative responses in four tone threshold conditions was compared between the two groups. The results showed a main response effect (*F(1,35)=69.782, p<.001, η²_p_=.666*) and threshold condition effect (*F(3,105)=17.289, p<.001, η²_p_=.331*) on the confidence rating. Although no significant main CH group effect (*F(1,35)=0.056, p=.815, η²_p_=.002*) or the interaction effects of CH group*condition (*F(3,105)=1.515, p=.215, η²_p_=.041*) or CH group*condition*response (*F(3,105)=5.468, p=.162, η²_p_=.055*) was observed, the results showed a significant interaction effect of CH group*response (*F(1,35)=4.382, p=.044, η²_p_=.111*). The post hoc pairwise comparisons showed that although the CH high perceiver group rated higher confidence in the positive response but lower confidence in the negative response, there was no significant group difference (positive:*t(35)=.819, p=.418*, negative:*t(35)=-1.642, p=.110*) (Fig. 2D).

Considering the unequal number of trials over the 12 blocks, we then investigated the group difference in the behavioural responses to CH perception over the 12 blocks, including positive response number at the 75% and 0% threshold conditions and confidence at the 0% threshold condition. The linear mixed-effect model revealed a main CH group effect (*F(1,35)=57.276, p<.001*), a main block effect (*F(11,385)=7.803, p<.001*), and an interaction effect of CH group*block on the FA number (*F(11,385)=5.284, p<.001*) (Fig. 2E). The post hoc pairwise comparisons showed consistent and larger FA responses for the CH high perceiver group after the third block. In contrast, there was no significant group effect on the positive response number at the 75% threshold condition (*F(1,35)=2.225, p=.145*) (Supplementary Fig. S1), ruling out the risk of response bias during the CH session. For the confidence rating at the 0% tone threshold, the linear mixed-effect model revealed a significant main effect of response (*F(1,805)=620.057, p<.001*) and an interaction effect of CH group*response the confidence rating. No block (*F(11,805)=1.454, p=.114*) and CH group effects (*F(1,35)=2.466, p=.125*) on the confidence rating or interaction effect of CH group*block (*F(11,805)=0.994, p=.450*) or CH group*block*response (*F(11,805)=1.022, p=.424*) was observed (Fig. 2F). These results are in line with those published by Powers and Corlett’s original study (Powers et al., 2017).

### 3.2 ERP and time–frequency results

**Fig. 3.**
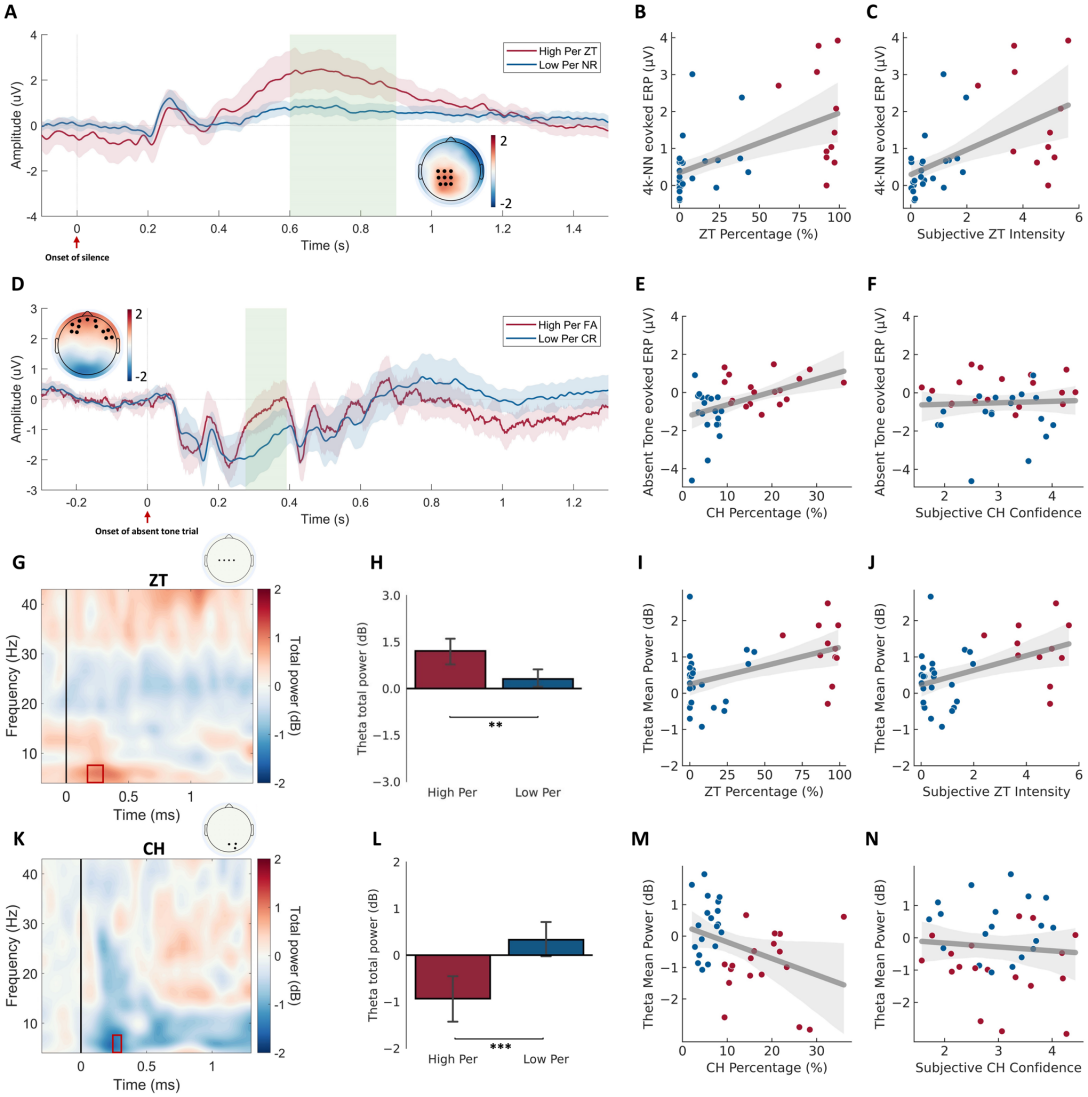
Neural representations for the two auditory illusions. ERP plot for the ZT (A) and CH illusions (D). The ERPs are grand averaged in each condition across the channels in black. Shaded regions represent the 95% CI of ERP for the illusion perception (i.e., ZT and FA) (in red) and non-illusion perception (i.e., NR and CR) (in blue) conditions. Green regions represent the time range that showed significant differences between the two groups. Topographical plot for the group difference. The mean amplitude was averaged across the time points that showed a significant difference between the two groups (regions in green). The channels of interest that showed significant differences are marked in black. Scatter plots for the correlation between the 4k-NN evoked ERP and ZT percentage (B), and ZT intensity rating (C). Scatter plots for the correlation between the absent tone evoked ERP and CH percentage (E), and CH confidence rating (F). Time–frequency representation for the group difference in the ZT (G) and CH (K) conditions. Time–frequency data were extracted and averaged from the channels labelled in the topographical plot. The box in red demonstrates the time–frequency representation that showed a significant difference between the two perceiver groups in individual illusion conditions (*p<.05*, two-tailed). Group comparison of the averaged total power across the significant time–frequency cluster in the ZT (H) and CH (L) conditions. Data are present with mean with the error bar of 95% CI. Scatter plots for the correlation between the theta power and ZT percentage (I), and ZT intensity rating (J). Scatter plots for the correlation between the theta power and CH percentage (M), and CH confidence rating (N).**p<.01.

We then investigated the neural measure of two auditory illusions. For the ZT session, there was no significant group difference after the onset of 4k-NN, but we observed a significant increase in the ZT-induced ERPs of the ZT high perceiver group between 600 and 900 ms after the offset of 4k-NN (*t_max_=8635.1, p=.002*), relative to the NR-induced ERPs in the ZT low perceiver group (Fig. 3A). This significant difference in amplitude was localized across central-parietal channels. The mean amplitudes of ERPs showed a significantly positive correlation with both the percentage of ZT perception (*r=.564, p<.001*) (Fig. 3B) and the intensity rating of ZT perception (*r=.559, p<.001*) (Fig. 3C), suggesting a consistent neural correlates of ZT illusion. For the CH, the FA-induced ERPs in the CH high perceiver group showed significantly increased amplitudes between 276 and 392 ms after the onset of absent trials (i.e., the visual checkboard was presented) (*t_max_=2577.2, p=.025*), compared with the CR-induced ERPs in the CH low perceiver group (Fig. 3D). This difference was lateralized to the left frontal region. The mean amplitude of ERPs showed a significantly positive correlation to the percentage of CH (i.e., FA rate) (*r=.445, p=.006*) (Fig. 3E) but no significant correlation to the subjective confidence (*r=.049, p=.773*) (Fig. 3F).

The time–frequency representations of the two auditory illusions were computed and compared between the high and low perceivers. We observed a significant increase in the theta frequency band between 168 and 296 ms after the offset of 4k-NN during the ZT perception, located in the central region (*t_max_=1464.5, p=.035*) (Fig. 3G), though no significant difference was identified between 600 and 900 ms. The post hoc comparison further supported a significant increase in the averaged theta power across the significant channel-time-frequency cluster for the ZT high perceivers (*t(35)=3.283, p=.002*) (Fig. 3H). The theta total powers were significantly correlated with the percentage of ZT perception (*r=.478, p=.003*) (Fig. 3I) and intensity rating of ZT perception (*r=.451, p=.005*) (Fig. 3J). For the CH session, compared with the CR condition in the CH low perceiver group, the CH high perceiver group showed a significant decrease in the theta frequency band between 242 and 310 ms after the visual checkboard in the FA condition was presented (*t_max_=-7590.6, p=.028*) (Fig. 3K). The significant group difference was located in the parietal–occipital region. The averaged theta power across the significant cluster of the CH high perceiver group showed a significantly decreased theta power relative to that of the CH low perceiver group (*t(35)=-3.940, p<.001*) (Fig. 3L). The averaged theta power also showed a significantly negative correlation to the percentage of CH (*r=-.372, p=.023*) (Fig. 3M) but no significant correlation to the subjective confidence (*r=-.039, p=.819*) (Fig. 3N).

### 3.3 Source localization

The induced neural activities by two illusions were further localised at the source level. The activities were averaged across the time of interest that showed significant differences between two conditions in each session and reconstructed for source signals. The results showed that the perception of two auditory illusions was linked with distinct activation of regions.

**Fig. 4.**
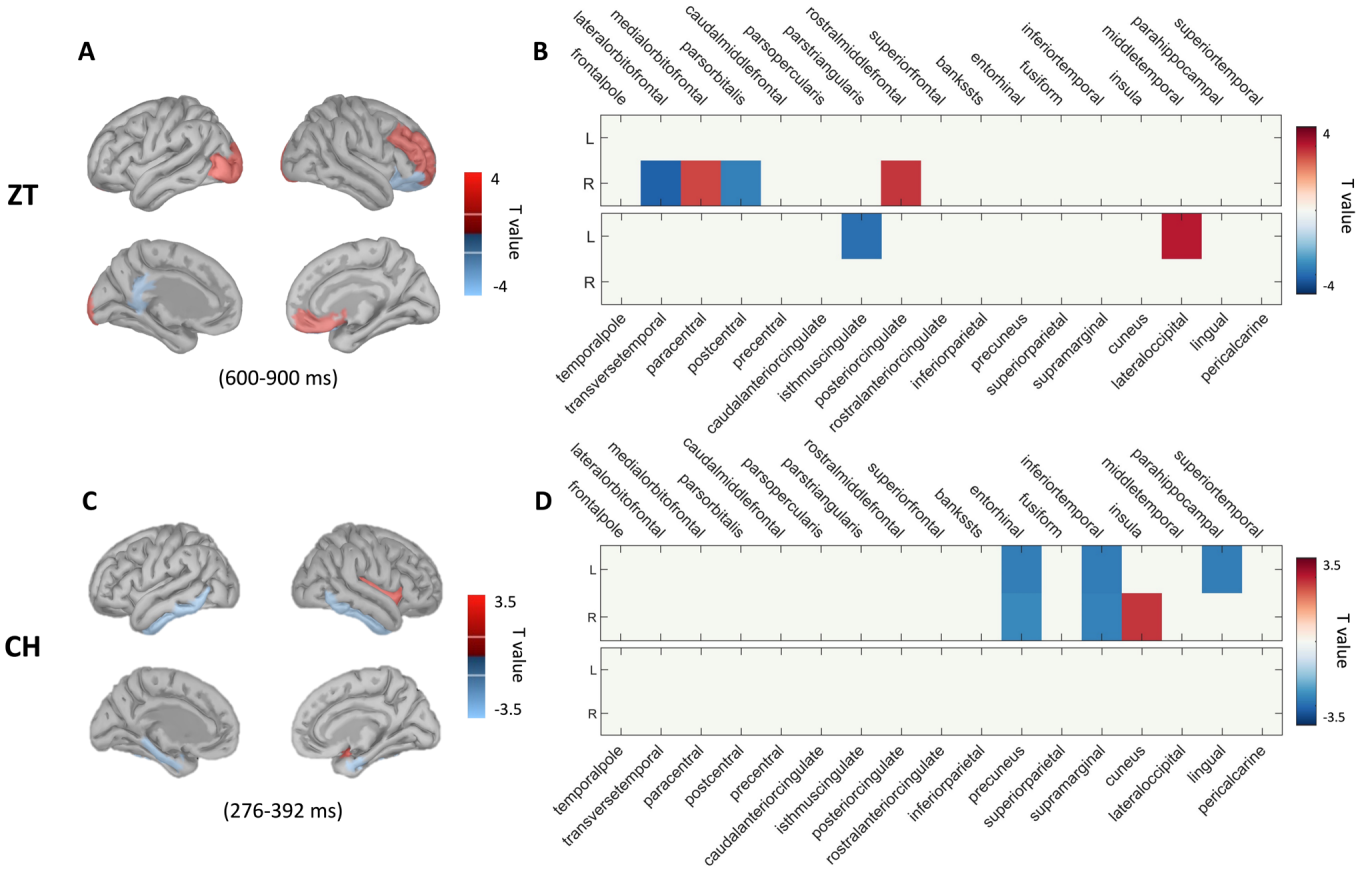
The results of source localization during the illusory perception. The left panel shows the absolute t-value of condition difference in the ZT session (A) and CH session (B) by the scout-based permutation test. Scouts were defined by the Desikan–Killiany Atlas. The mean activity of each scout was averaged across the time of interest from the ERP analysis. The regions that showed significant differences between the two trial conditions after correcting for multiple comparisons are shown (*p<.05*, two-tailed). The right panel demonstrates the t-value of the significant regions in the ZT session (C) and CH session (D). Boxes represent either significantly increased (in red) or decreased (in blue) activation in the illusion conditions than non-illusion conditions in individual sessions (*p<.025*, two-tailed).

Specifically, compared with the ZT low perceiver group, the ZT high perceiver group demonstrated significantly increased activity in the right medial orbitofrontal cortex around the medial prefrontal cortex (mPFC) (*t(35)=3.17, p=.002*), right rostral middle frontal gyrus around the prefrontal cortex (PFC) (*t(35)=3.528, p<.001*), and left lateral occipital sulcus (*t(35)=3.932, p=.002*). By contrast, there was significantly decreased activity in the right lateral orbitofrontal cortex (lOFC) (*t(35)=-4.061, p=.002*), right pars orbitalis (*t(35)=-3.413, p=.002*), and left isthmus of cingulate gyrus, close to the ventral posterior cingulate cortex (vPCC) (*t(35)=-3.736, p=.002*), left lingual gyrus (*t(35)=-3.868, p=.004*), and left pericalcarine cortex (*t(35)=-3.217, p=.004*) (Fig. 4A, B).

For the CH model, the FA in the CH high perceiver group was identified with significantly increased source activity in the right insula (*t(35)=3.548, p=.003*) relative to the CR condition in the CH low perceiver group. By contrast, there was significantly decreased activation in the bilateral entorhinal cortex (EC) (right:*t(35)=-3.221, p=.003,*left:*t(35)=-3.474, p=.003*), bilateral inferior temporal gyrus (ITG) (right:*t(35)=-3.375, p=.004,*left:*t(35)=-3.486, p=.002*), and left parahippocampal gyrus (PHC) (*t(35)=-3.443, p=.002*) (Fig. 4C, D).

### 3.4 Network-based approach

**Fig. 5.**
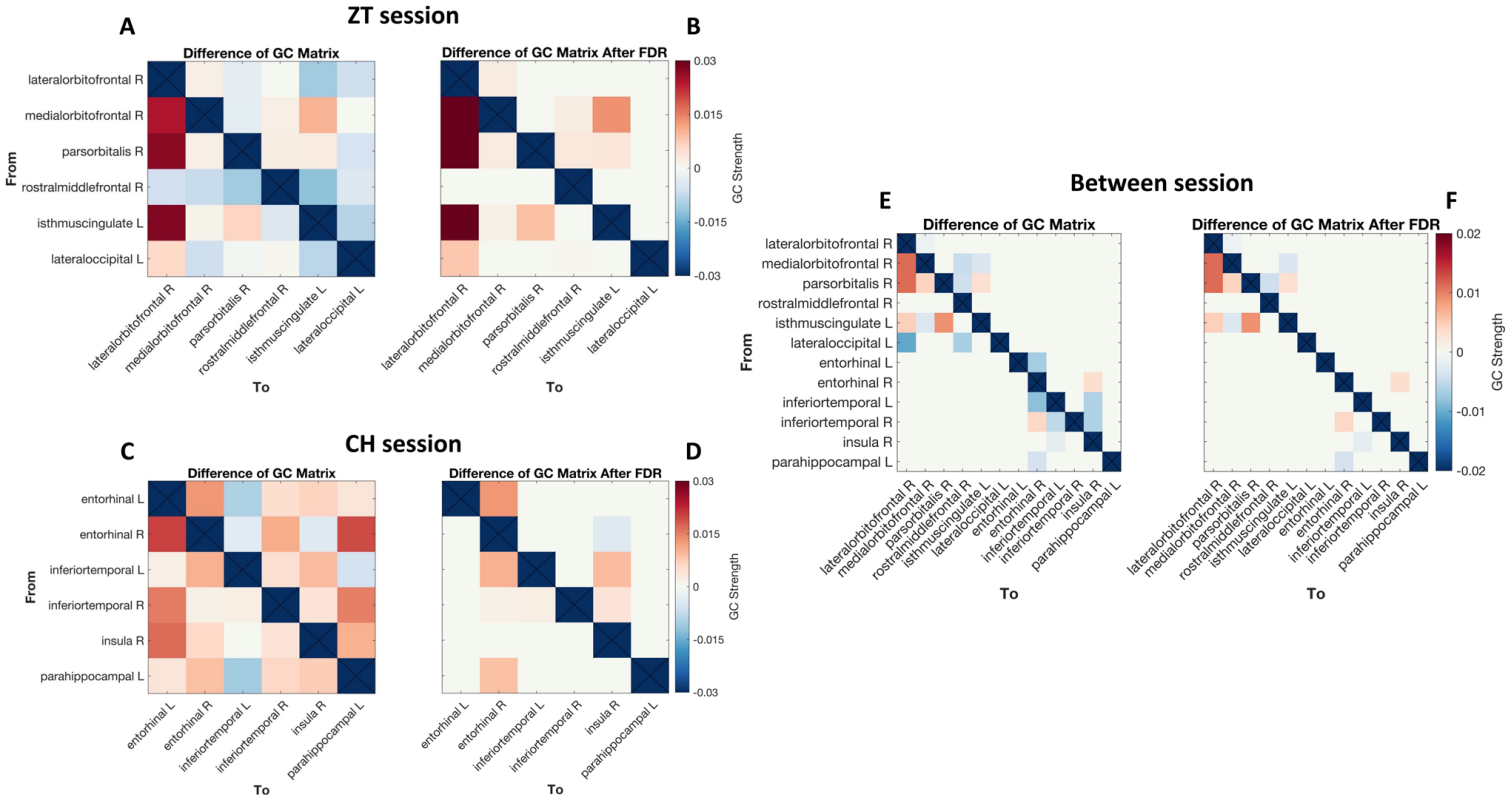
The directed connectivity of two auditory illusion networks. GC connectivity for the ZT network, with (A) showing the raw differences between the ZT and NR conditions and (B) showing significant connections after FDR correction. GC connectivity differences for the CH network, with (C) showing the raw differences between FA and CR conditions and (D) showing significant connections after FDR correction. Colour scales indicate GC strength differences: red represents stronger connections for the illusion condition, while blue indicates weaker connections. Direct comparison of GC connectivity between ZT and CH conditions, with (E) showing raw differences and (F) showing significant differences after FDR correction. Red represents stronger connections for the ZT condition, while blue indicates stronger connections for the CH condition.

We then computed the GC connectivity network for the illusion and non-illusion conditions for each session and analysed the connectivity difference between the two, taking a network approach to investigate the neural processes of two illusion models, respectively. For the ZT session, the connectivity pattern is shown inFigure 5A, B. Particularly, the strongest connectivity was observed to the right lOFC. Apart from this, the main receivers of the ZT network consisted of mPFC, PFC, and left vPCC, whereas the main senders were the right mPFC, right pars orbitalis, and left vPCC (Fig. 5B). Alternatively, the network of CH is shown inFigure 5C and D, in which the right EC and right insula received main information from the left PHC and/or the bilateral ITG (Fig. 5D).

In order to distinguish the signature network patterns of two illusions from the shared perceptual network, we directly compared the network connectivity between the two illusion conditions (Fig. 5E, F). The results showed that compared with the CH, the ZT network showed significantly stronger connections to the right lOFC, which was consistent with the connectivity results of within-ZT comparisons. Alternatively, the right pars orbitalis and left vPCC were the main senders. By contrast, compared with the ZT, the CH network showed significantly stronger connections from the left PHC to the right EC and from right insula to the left ITG. Interestingly, some connectivity in two illusion networks also showed stronger GC strength than those in the other networks. For the ZT, we observed stronger GC strength of the connectivity in the CH network from the right ITG to the right EC and from the right EC to the right insula than the CH. For the CH, we observed stronger CG in the ZT network from the right lOFC and left vPCC to the right mPFC, from right mPFC to the left vPCC and from the right pars orbitalis to the right PFC.

## 4 Discussion

This study, to the best of our knowledge, is the first to use two auditory illusion models in the same sample to contrast the neural mechanisms and neural networks of phantom perception driven by bottom-up or top-down factors. Auditory illusions give us a cost-effective and causal way of investigating the neural mechanisms of phantom perception, which is a characteristic clinical phenotype in several disorders such as tinnitus, schizophrenia, and different types of dementia. However, in a clinical population, studying the neural signatures of perception by itself is confounded by the disorder-related changes and other co-morbidities. In this regard, experimentally induced illusions give us a controlled environment to exclusively study the neural correlates of their perception.

In this study, we used the well-established ZT and CH paradigms that induce auditory illusions either as an after-image driven by a stimulus with a deficient sensory information (Norena et al., 2000) (i.e., bottom-up factors) or as a cued response to an absent stimulus driven by an internal model built through a learned association between an auditory target and a non-auditory cue (Powers et al., 2017) (i.e., top-down factors). The behavioural results of both these conditions are in line with previously published results. Furthermore, both factors hold significance in the clinical world as potential mechanisms that could trigger the perception of tinnitus. In this study, we used these models as proxies to understand the neural networks contributing to the perceptual and cognitive components associated with tinnitus.

### 4.1 Neural mechanisms of an auditory illusion driven by bottom-up factors

For the ZT illusion triggered by the temporary sensory deficit (i.e., broadband noise with a 1 octave frequency notch centred at 4000 Hz), we first observed increased theta power during the time of the expected after-image perception in the central region for those who reliably perceived the illusion compared with those who did not. Furthermore, the intensity and frequency of the illusion were both significantly correlated with the amplitude of the ERP and the strength of the theta/alpha power. These results are consistent with our previous study (Mohan et al., 2020) and complementary to the results from Leske and colleagues who showed that the strength of the illusion correlated with decreasing alpha power (Leske et al., 2014). The notched noise may lead to the imbalance between inhibitory and excitatory modulation at the cortical level and consequent disinhibited neuronal activities act as the sensory signals of ZT illusion (Leske et al., 2014). Furthermore, our results also align with the prior studies showing that auditory phantom perceptions, such as tinnitus, are often linked to changes in theta activity (De Ridder et al., 2015;Llinás et al., 1999). While this resemblance to tinnitus-related thalamocortical activity provides a compelling context, the present results specifically demonstrate how experimentally induced sensory deficits can drive oscillatory changes that correlate with the strength of the auditory illusion. Thus, this study may extend the role of ZT as a neuronal model of tinnitus (Norena et al., 2000;Pantev et al., 1999) to a more comprehensive model for understanding the neurophysiology of auditory phantom perception.

From a neural network perspective, we observe increased activity in the medial prefrontal cortex (mPFC), rostral middle frontal gyrus, and the occipital lobe and decreased activity in the orbital-frontal cortex (OFC), ventral posterior cingulate cortex (vPCC), and pars orbitalis. The activation of the mPFC, particularly the pregenual and subgenual parts, has been extensively studied in the phantom perception literature as the “gating system” or “noise-cancelling system” (Leaver et al., 2011;Mühlau et al., 2006;Rauschecker et al., 2010). This activation aligns with the compensatory mechanism in response to bottom-up sensory deficits, such as those induced by the notched noise in this study. Specifically, the temporary sensory loss might create prediction errors, which drive compensatory activity in higher-order regions, including the mPFC (Hullfish et al., 2019;Mohan et al., 2020). Increased activation, particularly in the theta band, has been shown to be a marker of phantom perception in the absence of hearing damage (Vanneste et al., 2019). The PFC especially the dorsal-lateral region is shown to be part of the auditory consciousness network (Brancucci et al., 2011) and particularly in processing pitch-related information (Adamchic et al., 2012). In addition, the vPCC is more connected with the medial temporal regions and involved with internally directed cognition (Leech & Sharp, 2014). In contrast, the deactivation of the OFC and pars orbitalis can be understood in light of their higher-order functions, as explained in the following section. Specifically, recent evidence suggested that the OFC contributes to generating predictions about perceptual events, including auditory stimuli (Asko et al., 2024). Lesions in the OFC have been associated with altered hierarchical auditory predictive processing, indicating its role in anticipating and interpreting auditory information (Asko et al., 2024).

The GC results also aligned with the top-down modulation of auditory phantom perception, with the core nodes of the network consisted of lOFC, mPFC, and vPCC. mPFC and PCC have been proposed to be the key nodes of the default mode network (DMN), which is activated during the resting state while being deactivated when a task-relevant network is involved in cognitive or perceptual function (Raichle, 2015). The dynamic process of DMN helps convey the narrative content of information (Baldassano et al., 2018), which is linked with high-level PE that is associated with the expectation of regularity in the current context or the environment. Indeed, the DMN has been demonstrated as a main hub for processing high-level PE (Brandman et al., 2021), reflected by its deactivation concurrent with the repetition of known-surprised events. Therefore, the connectivity within the DMN and between the DMN and lOFC might be a good indicator for the involvement of altered auditory predictive processing during the ZT perception: after the temporary sensory loss, the prediction errors of the sensory anticipation drive an active compensation that is perceived as the ZT illusion. This compensatory process highlights the causal relationship between bottom-up sensory deficits and top-down modulation by higher-order regions, such as the mPFC and DMN.

### 4.2 Neural mechanisms of an illusion driven by top-down factors

By contrast, for the CH illusion driven by strong internal expectation, the current study indicated a decrease in parietal theta power during the CH perception. Parietal theta oscillation is proposed to play a very important role in audiovisual encoding and multisensory integration. However, it still remains debatable how theta oscillation modulates this cognitive process. For instance, theta activity has been shown to positively correlate with the modulation of audio-visual integration during different cross-modal paradigms (Seijdel et al., 2024;Xie et al., 2021). Yet, parietal-occipital theta also showed an inversed correlation with the performance during an audio-visual associative learning task (Puszta et al., 2019). Our findings supported the inhibitory role of parietal theta oscillation in modulating multisensory integration and associative learning. As the power of theta band decreased, high perceivers falsely attributed the same source of auditory tone with the visual cue, leading to more FA perception. Nevertheless, more evidence is expected to cross-validate our findings and the role of parietal theta oscillation in modulating multisensory integration and associative learning.

From a network perspective, we observe increased activity in the insula and decreased activity in the EC, inferior temporal gyrus (ITG), and parahippocampal cortex (PHC) for the high perceivers compared with the low perceivers. The increased activity in the insula in the CH high perceivers is in line with the literature showing its involvement in the multisensory integration of auditory and visual stimulus (Protas, 2018) leading up to the establishment of a strong internal model which the CH high perceivers are hypothesized to base their perceptual decision making. Furthermore, the insula is also part of the salience network which is shown to be activated in the presence of a phantom percept that is both experimentally induced (Powers et al., 2017) and clinically present (Chen et al., 2023). The involvement of the temporal and medial temporal lobe in multisensory integration is not fully understood. However, one study suggested (Sun et al., 2024) that an inactivation of the EC could result in the failure to integrate audio-visual information which may be the case for CH low perceivers. In addition, the perception of auditory hallucination was linked with the deactivated PHC (Diederen et al., 2010). Since the PHC plays a central role in memory recollection (van Strien et al., 2009), the disinhibition from deactivated PHC might indicate an erroneous information retrieval and hence to incorrect responses, namely the CH illusion.

The GC results emphasized main information flow from the PHC and ITG to the EC and insula, further supporting altered top-down networks modulating multisensory association. The PHC and EC are widely involved in the memory network. The PHC prevents irrelevant signals from the hippocampus as well as provides the temporal and spatial information that is going to be encoded into long-term memory (Aminoff et al., 2013). The EC is involved in the contextual processing that helps associate multiple sensory modalities to identify an object (e.g., clucking/growling (auditory) with hen/tiger (visual)) (Bar, 2004;Bar & Aminoff, 2003). Alternatively, insula is important for auditory processing (Bamiou et al., 2003). The connectivity from the ITG has been linked with the top-down modulation of repetitive perception to facilitate responses and task performance (Buckner et al., 2000). Thus, the connectivity from the PHC and ITG to the EC and insula may indicate the involvement of the auditory memory in driving the perceptual CH illusion. Taken together, these findings suggested a high-order capability to utilize internally generated perspective from one sensory modality (i.e., visual cue) to shape our perception in another sensory modality (i.e., the CH illusion).

### 4.3 Comparing the neural mechanisms and networks of two auditory illusions

One of the most intriguing findings in this study is the distinct time–frequency patterns between the bottom-up (i.e., increased theta power) and top-down (i.e., decreased theta power) driven auditory illusions, implicating the hallmark of difference in their underlying neural mechanisms. For the ZT illusion, while it is driven by the manipulated noise stimuli, the increased theta activity might indicate an involvement of top-down processes in its neural mechanism. Central theta activity typically links with the cognitive processes when attention is directed to relevant sensory information or when there is a need to suppress a response to non-relevant stimuli (Cohen & Donner, 2013). Therefore, the increased theta power during the ZT perception could be an indicator of attentional modulation to the illusory perception. By contrast, the parietal theta oscillation is characterized with top-down processing such as multisensory integration and associative learning, as discussed above. The reduction in theta power might sign a different mode of top-down control, where the cognitive effort to integrate or associate the stimuli eventually elicits the perception of FA.

Furthermore, the distinction in neural networks further emphasize the complexity of phantom perception. We observed the stronger network connectivity mainly from pars orbitalis and vPCC and mainly to the lOFC during the ZT illusion, relative to the CH illusion. These connections might be associated with generating and reconciling prediction errors in response to temporary sensory deficit, highlighting a compensatory mechanism of the ZT illusion where the involved networks dynamically adapt to altered auditory inputs by endogenous predictions. In contrast, the connections from left PHC to the right EC and from right insula to the left ITG in the network were stronger for the CH illusion relative to the ZT illusion. These findings might emphasize the roles of memory modulation and multisensory integration to construct an internally driven perceptual illusion. Particularly, the PHC-EC connection underscores the role of memory networks in contextual and associative processing, pointing out a mechanism that relies heavily on internally generated expectations to shape the auditory illusion.

Taken together, the time–frequency results might emphasise a fundamental divergence in how bottom-up and top-down processes contribute to the perception of these two auditory illusions, whereas the contrast in the network connectivity deepens our understanding of the distinct dynamic network interplay between attention, memory, and sensory integration during the auditory phantom perception.

### 4.4 The relevance of two auditory illusions to tinnitus mechanism

The relationship among tinnitus, ZT, and CH was evident. For instance, it has been proposed that the mechanisms of ZT and tinnitus are closely connected (Hoke et al., 1996;Mohan et al., 2020). Hearing loss is the most common reason for tinnitus through cochlear damage. The notch noise for generating ZT, similarly, is believed as a temporary model of sensory loss in the hair cells (Schilling et al., 2021). One previous study pointed out that people with tinnitus are also more likely to perceive ZT, though the degree of hearing loss of subjects was not reported (Parra & Pearlmutter, 2007). Apart from the neuronal model,Hullfish et al. (2019)also proposed the maladaptive predictive coding as the neural mechanism that underlies both ZT illusion and tinnitus, in which the missing sensory information creates prediction errors that drive the phantom perception as a compensation to minimise prediction errors. The increased theta oscillation was evident in auditory and non-auditory regions (e.g., PHC, pgACC/vmPFC, OFC) during both ZT perception and tinnitus perception in numerous studies (Mohan et al., 2020;Vanneste et al., 2024;Weisz et al., 2005,^2007^). Bridging this to clinic, theta-burst stimulation, a patterned form of brain stimulation that mimics rhythmic bursts of 3–8 Hz endogenous brain rhythms, has been identified with a potential to suppress tinnitus perception in several sham-controlled studies (Chung et al., 2012;Forogh et al., 2014;Lorenz et al., 2010), though it is not consistent with the negative results in other studies (Plewnia et al., 2012;Schecklmann et al., 2016). Overall, this evidence supported the view that the neural mechanisms of Zwicker tone and tinnitus with hearing impairment can be overlapped. Theta oscillations may play a role in synchronising activity across auditory and non-auditory brain regions, which may contribute to phantom perception in both physiological and pathological conditions.

Alternatively, by using the CH, the mechanism of tinnitus was further explored. More specifically, while patients with tinnitus showed no significant difference in behavioural or neural responses to the auditory CH, they were more likely to perceive visual CH compared with healthy controls (Yasoda-Mohan et al., 2024). These results suggested that those who are more likely to perceive CH are more likely to build a strong perceptual model for the current environment and shape the perception based on the prior expectation. This aligns with our results that high CH perceivers showed higher proneness to perceive the CH illusion. By contrast, for tinnitus patients, there may be a rivalry between the existing tinnitus perception and the induced auditory CH percept, whereas the higher CH rate in the visual domain suggested their domain-wide proneness to build stronger prior models.

From the perspective of tinnitus, the above results show that both paradigms have the potential to serve as models for the perceptual component of tinnitus. The ZT model focuses on the bottom-up factors that generate tinnitus such as sensory deficit, increased central gain, and dysfunction of “noise-cancelling” system. Alternatively, this is the first study, to the best of our knowledge, that proposes the potential role of the CH model as a tinnitus model to investigate the top-down and cognitive modulation of tinnitus, such as relationship of the auditory domain with other sensory modalities such as vision and somatosensory, adaptability of the internal model to changes in external stimulus and effect of learning the association between auditory components with non-auditory cues. These distinctions may offer a basis for future research to explore whether different types of tinnitus are more aligned with ZT-like or CH-like mechanisms, or whether both paradigms represent coexisting processes across the spectrum of tinnitus cases.

### 4.5 Limitations and future implication

There are some limitations that need to be taken into consideration. First, in the ZT session, participants measure the loudness of the afterimage effect by self-controlled timing. This prevents us from investigating and distinguishing the post-perceptual components, for example, decision-making and/or response preparation, from the perceptual ERPs. Future studies should include reaction time analyses and correlate with neural and behavioural measures (e.g., intensity and confidence) to clarify their contributions to ERPs. While our findings provide robust evidence supporting the reliability of ZT perception, the significantly higher illusory responses in the WN condition among the high ZT perceiver group compared with the low ZT perceiver group indicate a potential risk of response bias. This raises the possibility that factors such as heightened suggestibility, attentional focus, or predispositions to perceptual inference could have influenced the results. While we controlled for reliability during participant screening, further studies are recommended to apply measures such as signal detection theory metrics to rule out the effect of response bias from true perceptual effects.

Meanwhile, considering the heterogeneity of tinnitus in terms of its subjective characteristics and aetiology (Cederroth et al., 2019;Mohan et al., 2022), the auditory illusion models used in this study cannot fully represent the complexity of tinnitus or serve as general models for all subtypes of tinnitus. More specifically, neither of the two illusion models involve emotional components, though the negative impact of psychological factors (i.e., depression, stress) on the generation or deterioration of tinnitus is evident across studies. Furthermore, each illusion model in the study operates only within a specific framework. The ZT illusion could act as a “reversible tinnitus model” that is driven by high-frequency sensory deficit, whereas the CH, as a top-down driven illusion, may provide insights into cognitive mechanisms of tinnitus. Alternatively, other auditory illusion models have been developed to investigate different mechanisms of tinnitus. For example,Job et al. (2016)used click-trains to generate tinnitus-like tonal aftereffects, hypothesizing peripheral influences in the middle ear and neural correlates in the somatosensory cortex (Job et al., 2016). Therefore, these models highlight the diverse approaches available to explore the complex neural and perceptual processes underlying tinnitus and provide complementary insights into its multifaceted nature.

In terms of the methodology, the smaller sample size than the estimated sample size may potentially reduce the ability to detect smaller effects. As a result, some findings should be interpreted with caution, particularly at the source level. Also, the standard head model was applied for each subject to compute the source localization rather than the individual MRI models, which is at risk of dampening the precision of source estimation (Brodbeck et al., 2011). In addition, recent research also raised concerns about the consistency of effectivity connectivity in EEG studies, which was related to the choice of the inverse method and source imaging package (Mahjoory et al., 2017). Future research is expected to validate the current findings through other neuroimaging techniques and analysis. Furthermore, the linear GC method may not fully capture the transmission of nonlinear information or the functional roles of regions in brain networks. This is especially relevant where nonlinearities play a significant role, such as the coupling of neural population activities or the nonlinear relationships between physiological parameters and their observable effects (Stephan et al., 2008). Thus, further research is recommended to apply nonlinear GC measures, such as Kernel Granger causality (Liao et al., 2009) or Local Linear Nonlinear Autoregressive models (Freiwald et al., 1999).

Non-invasive neuromodulation techniques, such as transcranial direct current stimulation (tDCS) and transcranial magnetic stimulation (TMS), are encouraged to target the related networks during auditory illusions, which could provide more direct evidence of their roles in modulating the perception of phantom perception (Langguth et al., 2012;Song et al., 2012). Furthermore, in this study, we observed a significant difference in confidence in perceptual decisions between the two CH perceiver groups. Despite no significant correlation between CH evoked or induced activity and confidence rating, it suggests the potential interaction between confidence and perceptual experience. Indeed, recent evidence implicated perceptual confidence as a reinforcement signal for perceptual learning in the absence of external feedback, which could be involved in establishing and strengthening prior knowledge and expectation under the predictive coding framework (Corlett et al., 2019). Further research is expected to investigate the role of confidence in perceptual decision-making and phantom perception.

## 5 Conclusions

Through the two auditory illusion models, the neural correlates and networks of phantom perception induced by either bottom-up or top-down factors were causally distilled from this study. The immediate effect of the sensory deficit was modelled by the ZT illusion through manipulated stimuli, whereas the CH illusion results from the modulation of strong expectations due to the association between auditory and visual inputs. Distinct time–frequency representations differentiated the two illusions, with increased central theta activity during ZT perception and decreased parietal theta activity during CH perception. The network analysis further revealed unique connectivity patterns, linking ZT to default mode network and predictive processing system and CH to top-down modulation of memory and sensory regions. These findings could serve as important clues of the neural architecture that drives broader perspectives on the physiology of perception and pathology of perceptual disorder, such as tinnitus.

## Supplementary Material

Supplementary Material

## Data Availability

The data and code are available with the corresponding author. They will be shared on email request. The anonymized data after removing the identifying personal information will be provided.
