# Supplementary material for: Auditory illusory models as proxies to investigate bottom-up and top-down neural networks of phantom perception
Source: Imaging Neurosci (Camb). 2025 May 9;3:imag_a_00574. doi: 10.1162/imag_a_00574 (PMC12319843; doi:10.1162/imag_a_00574)
Supplement: Supplementary Material [file imag_a_00574-supp.pdf]

Supplementary Table 1. Trial number for ZT and CH comparisons

Sub ID	4k-NN ZT	4k-NN NR	Group by ZT	FA	CR	Group by CH
1	92	8	High ZT	62	223	High CH
2	0	100	Low ZT	41	230	High CH
3	0	100	Low ZT	27	259	High CH
4	0	100	Low ZT	8	278	Low CH
5	2	98	Low ZT	21	265	Low CH
6	97	3	High ZT	44	244	High CH
7	86	14	High ZT	30	258	High CH
8	24	76	Low ZT	46	241	High CH
9	92	8	High ZT	12	266	Low CH
10	8	82	Low ZT	27	260	High CH
11	98	2	High ZT	10	269	Low CH
12	92	8	High ZT	16	264	Low CH
13	38	62	Low ZT	23	265	Low CH
14	16	84	Low ZT	97	172	High CH
15	0	100	Low ZT	30	247	High CH
16	1	99	Low ZT	25	262	Low CH
17	95	5	High ZT	10	278	Low CH
18	0	100	Low ZT	20	231	Low CH
19	0	100	Low ZT	10	256	Low CH
20	0	100	Low ZT	16	272	Low CH
21	99	1	High ZT	32	255	High CH
22	43	57	Low ZT	61	219	High CH
23	0	100	Low ZT	6	278	Low CH
24	62	38	High ZT	65	214	High CH
25	23	77	Low ZT	39	235	High CH
26	39	61	Low ZT	14	272	Low CH
27	0	100	Low ZT	23	254	Low CH
28	1	99	Low ZT	16	267	Low CH
29	87	13	High ZT	51	237	High CH
30	0	100	Low ZT	21	245	Low CH
31	2	98	Low ZT	12	259	Low CH
32	97	3	High ZT	74	186	High CH
33	0	100	Low ZT	58	226	High CH
34	0	100	Low ZT	18	263	Low CH
35	8	92	Low ZT	18	216	Low CH
36	2	98	Low ZT	75	212	High CH
37	1	99	Low ZT	59	227	High CH

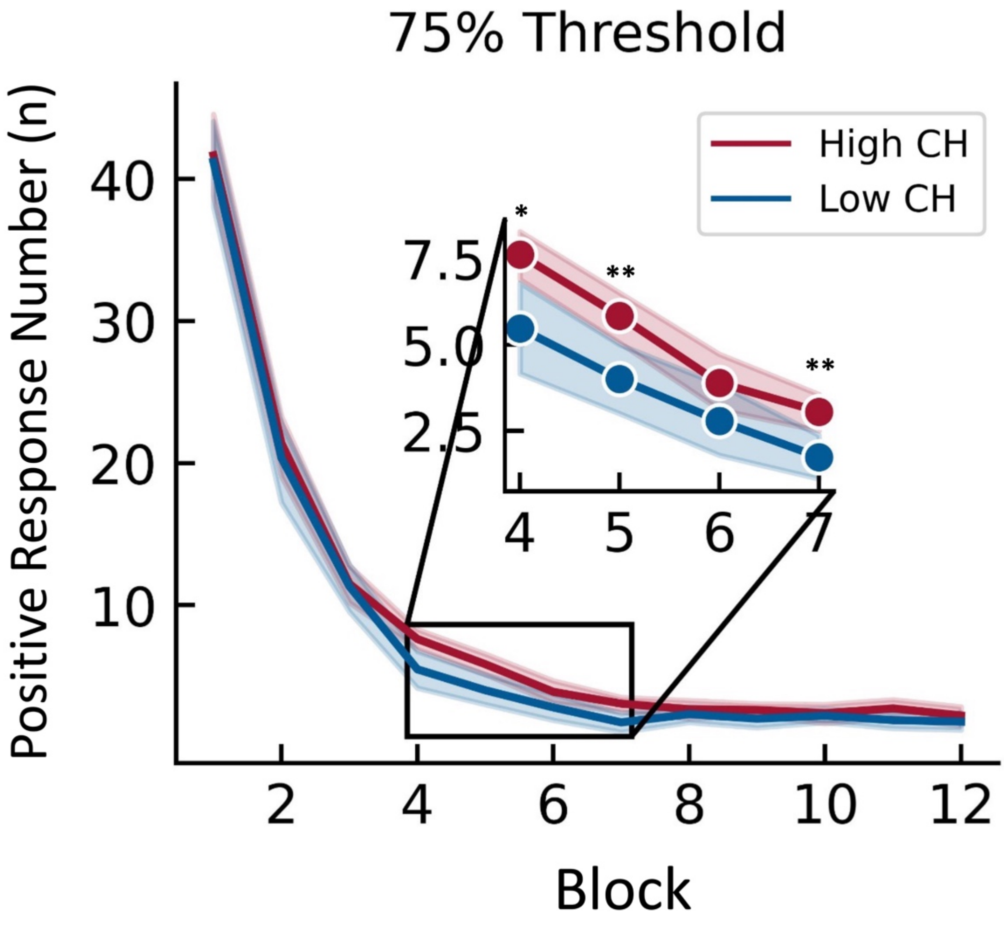

Supplementary Figure 1. Group difference of the positive response number in the 75% threshold condition over the blocks between two CH groups. *$*p < .05$ $**p < .01$.*
